# Structural variability of dyads relates to calcium release in rat ventricular myocytes

**DOI:** 10.1038/s41598-020-64840-5

**Published:** 2020-05-15

**Authors:** Marta Novotová, Alexandra Zahradníková, Zuzana Nichtová, Radoslav Kováč, Eva Kráľová, Tatiana Stankovičová, Alexandra Zahradníková, Ivan Zahradník

**Affiliations:** 1 Department of Cellular Cardiology, Institute of Experimental Endocrinology, Biomedical Research Center, Slovak Academy of Sciences, Bratislava, Slovakia; 2 Department of Muscle Cell Research, Institute of Molecular Physiology and Genetics, Centre of Biosciences, Slovak Academy of Sciences, Bratislava, Slovakia; 3 Department of Pharmacology and Toxicology, Faculty of Pharmacy, Comenius University in Bratislava, Bratislava, Slovakia

**Keywords:** Ion transport, Cardiovascular biology

## Abstract

Cardiac excitation-contraction coupling relies on dyads, the intracellular calcium synapses of cardiac myocytes, where the plasma membrane contacts sarcoplasmic reticulum and where electrical excitation triggers calcium release. The morphology of dyads and dynamics of local calcium release vary substantially. To better understand the correspondence between the structure and the functionality of dyads, we estimated incidences of structurally different dyads and of kinetically different calcium release sites and tested their responsiveness to experimental myocardial injury in left ventricular myocytes of rats. According to the structure of dyads estimated in random electron microscopic images of myocardial tissue, the dyads were sorted into ‘compact’ or ‘loose’ types. The calcium release fluxes, triggered at local calcium release sites in patch-clamped ventricular myocytes and recorded by laser scanning confocal fluorescence microscopy, were decomposed into ‘early’ and ‘late’ components. ANOVA tests revealed very high correlation between the relative amplitudes of early and late calcium release flux components and the relative occurrences of compact and loose dyads in the control and in the injured myocardium. This finding ascertained the relationship between the structure of dyads and the functionality of calcium release sites and the responsiveness of calcium release sites to physical load in cardiac myocytes.

## Introduction

Dyads of cardiac muscle cells are intracellular synapses that transmit excitatory signals from the plasmalemma to the juxtaposed terminal cisternae of sarcoplasmic reticulum, i.e., from the surface into the volume of myocytes. Generally, the basic requirement for myocyte contraction is an increase of cytosolic concentration of calcium ions^1^. In simple or small myocytes, such as in embryonic myocardium, the influx of calcium ions through plasmalemma activated by incoming action potential during systole may be sufficient^2^. However, cardiomyocytes of adult myocardium have to produce a substantial force. To achieve that, the cytosolic calcium increase is augmented by calcium release at numerous dyads^1^. The dyads, operating on the calcium-induced calcium release principle^3^, allow graded release of calcium ions from the lumen of the sarcoplasmic reticulum, where it had been accumulated during the diastole. In thin myocytes, such as those of atrial tissue, dyads occur mostly at the myocyte surface^4^. In thicker myocytes of ventricular tissue, the plasmalemma develops a network of thin tubules, through which it transmits excitation to terminal cisternae of sarcoplasmic reticulum and thus forms numerous dyads very close to contractile myofibrils and in the whole volume of myocytes^4,5^.

The intensity of calcium release is controlled at the level of dyads by calcium signalling between DHPR and RyR (ryanodine receptor type 2) channels^6–9^. It is reasonable to expect that calcium release depends to a certain extent on the structure of the dyad, since the latter defines the spatial disposition between DHPR and RyR channels^10–14^ and thus the probability of RyR opening^8,15,16^ by calcium ions. The volume density of dyads is about one per cubic micrometre^17^, which translates to several tens of thousands of dyads per typical ventricular myocyte. Dyads differ in size, shape, and localization. Their size ranges from tens to hundreds of nanometres^18–21^. The smaller ones are more frequent than the large ones^19–22^. The number of RyRs per dyad varies from a few to hundreds^18–22^. Details of dyad structure in 3D were revealed with the use of electron tomography^22,23^. However, the variability in structural appearance of dyads has not been described yet. Most published images of dyads were obtained by transmission electron microscopy of ultrathin sections (<100 nm) of tissue samples, where the dyads are not seen in full. Typical images show dyads sectioned randomly, since they do not have a preferential spatial orientation. In such sections, the structural appearance of dyads varies substantially, although retaining the key morphological determinants, namely the profile of the t-tubule, of the terminal cisterna, and of RyRs in the junctional gap.

The structure and abundance of dyads varies during the myocyte lifetime^24^. Studies on animal models of heart failure and on human samples reported reduction of the t-tubule content^25–30^, as well as vesiculation^31^, dilation^11,32^ or overall reorganization of t-tubules^12,33,34^. T-tubule remodelling was shown to begin before echocardiographically detectable left ventricular (LV) dysfunction and to progress during structural changes in heart failure^35^. Disorganization of t-tubules was observed to lead to an increase in orphaned RyRs, to a loss of local control of Ca^2+^-induced Ca^2+^ release, and to reduced synchrony of Ca^2+^ release^35^. It was shown that small changes in the geometry between DHPRs and RyRs in dyads of cardiomyocytes of failing human heart may have profound effects on the ability of a DHPR to activate adjacent RyRs^11,27^. Disruption of t-tubule architecture in diseased cells of failing human heart was associated with a slight loss of RyR clusters^12^ and reduced co-localization between RyRs and DHPRs^27^.

All these facts endorse the opinion that properties of dyads are central to the quality of cardiac excitation-contraction coupling; however, the relationship between their structure and function is not well understood since simultaneous observation of the structure and the function of a dyad is not experimentally feasible. Therefore, we relied on the correlation of the variability of structural and functional indicators of dyads in correlated experiments. We hypothesised that specific structural features of dyads, including changes in qualitative morphological traits of t-tubule, dyadic gap, and terminal cisterna could be reflected in changes of calcium current and calcium release flux. The relation between structure and function of dyads was challenged by experimentally induced myocardial injury that was expected to induce adaptation response at the level of cardiac myocytes. We expected to observe differences in the dyadic structure that would correlate with differences in characteristics of calcium release of the two myocardial groups.

## Results

The structural and the functional variability of dyads was analysed in working myocytes of control rat myocardia (CTR) and of injured rat myocardia (IMY). We used the transmission electron microscopy to collect images of dyads, and the whole-cell patch-clamp combined with laser-scanning fluorescence confocal microscopy to collect records of calcium currents and of calcium spikes. Both the structural and the functional data were collected by random sampling from well-defined localities of myocytes to allow application of statistical methods of comparison.

We employed the method of myocardial injury based on a single administration of a high dose of isoproterenol to influence the structure of dyads by enforced remodelling of myocytes. Efficiency of the injury was verified after 15 days of recovery from the treatment at the whole heart level *in vivo* and *ex vivo*, as well as at the level of working myocytes (Supplemental Results). The compromised function of IMY hearts, measured *in situ* as well as *ex vivo*, was manifested by electrical imbalance and decreased systolic pressure developed by left ventricles (Supplemental Table S1). Morphological and functional characteristics of IMY myocardia corroborated the partial loss of contractile tissue and the onset of early phase of hypertrophy observed in the working tissue (Supplemental Results). These data confirmed that the isoproterenol treatment caused partial injury and remodelling of the heart muscle tissue, as intended (Supplemental Fig. S1).

### Structure of dyads varies in cardiac myocytes

**Figure 1:**
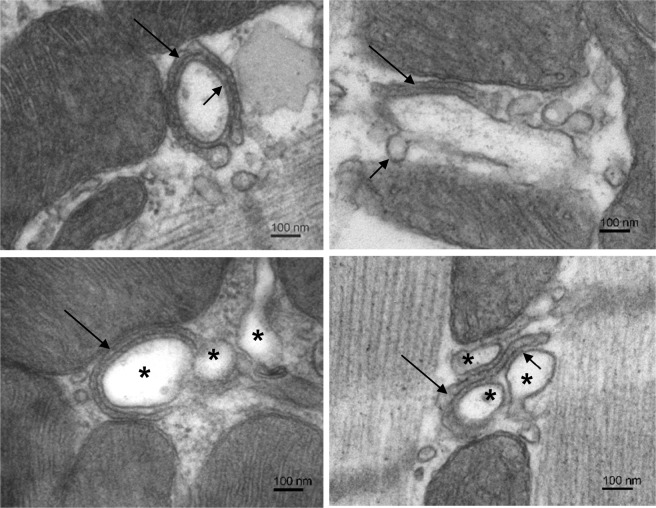
Compact dyads. Electron micrographs selected to illustrate structural variability of the compact type of dyads. Top left: A dyad with the t-tubule tightly surrounded by a flat cisterna containing condensed calsequestrin (long arrow). Numerous RyRs are present in the regular dyadic gap (short arrow). Top right: A dyad containing a t-tubule with budding caveolae-like formations (short arrow). A short section of the flat cisterna (long arrow) closely adheres to the t-tubule membrane. Bottom left: A dyadic microdomain with three t-tubule profiles (*). The narrow cisterna closely adheres to the t-tubule (arrow). Numerous RyRs cross the dyadic gap. Bottom right: A dyad made of 3 t-tubule profiles (*) and a narrow cisterna (long arrow) forms multiple tight dyadic gaps with numerous RyRs (short arrow). Note: The left and the right columns show images taken from control and injured myocardia, respectively, but they represent characteristic dyads of both experimental groups.

**Figure 2:**
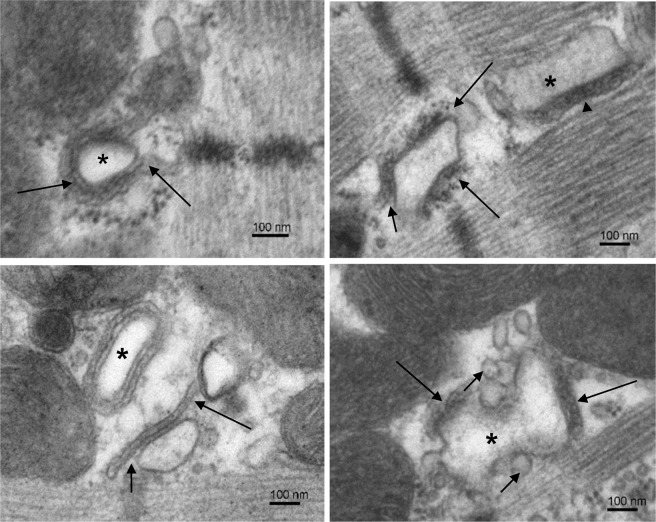
Examples of loose dyads. Electron micrographs selected to illustrate structural variability of the loose type of dyads. Top left: A dyad with a regular t-tubule (*) surrounded by a cisterna filled with homogenous electron-dense material and extending away from the t-tubule (arrows). Top right: Two dyads in a microdomain. The loose dyad between Z-lines has the junctional surface of cisterna partially deflected from the t-tubule (short arrow) and RyRs exposed to cytosol (long arrows). The compact dyad between A-bands of myofibrils contains a part of the longitudinal t-tubule (*) with well adherent cisterna (arrowhead). Bottom left: Two dyads in a microdomain. The dyad indicated by arrows is of the loose type because the junctional membrane of its cisterna deflects (short arrow) and extends (long arrow) from the t-tubule. The dyad made of regular t-tubule profile (*) is of the compact type of dyads. Bottom right: A dyad made of two cisternae (long arrows) and a deformed t-tubule (*). Numerous vesicles are present near the t-tubule (short arrows). Note: The left and the right columns show images taken from control and injured myocardia, respectively, but they represent characteristic dyads of both experimental groups.

The structure of dyads was analysed in electron microscopic images. Dyads could be clearly identified according to their typical appearance and preferential location in dyadic microdomains near Z-lines of myofibrils. The structural analysis was focused on dyadic t-tubules, terminal cisternae of sarcoplasmic reticulum and the gaps between them. To obtain a whole spectrum of dyadic substructures, a robust data set was collected by inspecting over 960 sarcomeres of each experimental group that consisted of 749 images of the control and of 707 images of the IMY dyads. The morphology of dyadic substructures varied broadly (see Figs. 1 and 2); however, structural traits characterizing the dyadic constituents were the same in both experimental groups. No new structural variant of dyads or their components was observed in the myocytes of injured myocardia, and *vice versa*, no structural variant of dyads observed in control myocytes was missing in IMY myocytes. Moreover, a broader inspection of many myocytes of both groups revealed that all structural variants of dyads could be found in any individual myocyte. Therefore, it was concluded that the morphological analysis did not reveal specific changes in dyadic forms that could be claimed responsible for differences in calcium release between the experimental groups.

Since the structure of dyads was unresponsive to the experimental injury, we hypothesised that the changes in calcium release arise from the changed population of structural variants of dyads. For the sake of simplicity and for comparison with functional results described below, the identified dyads were assigned to one of two basic structural types. We dubbed them as the compact and the loose dyads. As compact dyads were considered those dyads that showed their cisterna adhered tightly to the t-tubule membrane, with flat lumen containing condensed calsequestrin, and the ryanodine receptors localized only within the dyadic gap (Fig. 1). These included structures with a single cisterna and a single t-tubule profile (Fig. 1 top left), as well as ones with multiple t-tubule profiles (Fig. 1 bottom), or ones with discontinuities in t-tubule profiles due to the incidence of membrane protrusions (Fig. 1 top right). As loose dyads were considered those showing compromised adherence to t-tubules, often accompanied by major irregularities in terminal cisternae, such as lack of condensed calsequestrin, increased lumen volume, vesiculation, and misplaced RyRs (Fig. 2). Typically, cisternae of loose dyads adhered to t-tubules only partially, their membrane was partially deflected from the t-tubule or extended away from the t-tubule (Fig. 2 left), and some RyRs were exposed to the cytosol (Fig. 2 top right). Dyads with disorganized or irregular structure of both the t-tubules and the cisternae, with fragmented or vesiculated cisternae attached meagerly to their t-tubule (Fig. 2 bottom right), were also included in the loose group.

### Dyadic population reacts to myocardial injury

The average number of dyads per sarcomere, independent of the type, was essentially the same in both experimental groups (Table 1). Since neither the number of calcium release sites per sarcomere was different between the groups (Table 2, CRS density), the overall occurrence of dyads should not be a factor related to the observed functional changes. However, myocardial injury substantially changed the relative occurrence of the two dyad types (Table 1). In control hearts, the compact dyads clearly dominated over the loose dyads. In injured hearts, however, the relative occurrence of the compact and the loose dyads was almost the same. This finding indicates that the myocardial injury substantially changed the quality of dyadic population in working myocytes. The question was whether the change in the quality of dyads relates to changes in the calcium release function.

**Table 1:** Dyad density in cardiac myocytes.

	CTR	IMY	P
**Counts per sarcomere**			
All dyads	0.78 ± 0.02	0.73 ± 0.02	0.06
Compact dyads	0.58 ± 0.02	0.39 ± 0.03	4.0E-7
Loose dyads	0.20 ± 0.02	0.35 ± 0.03	1.8E-4
**Fraction of dyads**			
Compact dyads	0.74 ± 0.02	0.53 ± 0.04	3.6E-6
Loose dyads	0.26 ± 0.02	0.47 ± 0.04	3.6E-6

Data are given as mean ± s.e.m., n = 32 for both animal groups; P - Student’s t-test p-value.

**Table 2:** Parameters relevant to calcium signalling.

	CTR	IMY	P
**Whole-cell patch-clamp measurements**			
J_Ca_ (pA/pF)	7.14 ± 0.66	7.09 ± 0.57	>0.5
TTP (ms)	5.23 ± 0.15	5.03 ± 0.27	>0.5
FDHM (ms)	7.77 ± 0.25	7.35 ± 0.18	0.18
V_1/2_ (mV)	–18.4 ± 0.6	–16.2 ± 0.9	0.08
V_r_ (mV)	55.7 ± 1.1	52.9 ± 1.4	0.14
**Parameters of calcium spikes**			
CRS density	0.76 ± 0.02	0.73 ± 0.02	>0.5
Amp (ΔF/F_0_)	0.86 ± 0.02	1.00 ± 0.02	6.7E-7*
Lat (ms)	4.60 ± 0.21	5.56 ± 0.20	6.0E-9*
TTP (ms)	4.47 ± 0.10	5.48 ± 0.12	8.1E-10*
FDHM (ms)	8.43 ± 0.16	10.07 ± 0.16	1.3E-13*
**Parameters of integral calcium release flux**			
Amp (ΔF/F_0_)	0.56 ± 0.02	0.63 ± 0.03	0.060
Lat (ms)	3.05 ± 0.11	3.64 ± 0.13	0.002
TTP (ms)	6.27 ± 0.14	7.82 ± 0.29	6.0E-5
FDHM (ms)	9.77 ± 0.24	11.97 ± 0.45	0.010*

Data are given as mean ± s.e.m. estimated from 15 CTR and 17 IMY myocytes, except for V_1/2_ and V_r_ estimated from 5 CTR and 6 IMY myocytes. Parameters of calcium spikes were estimated from 218 CTR and 240 IMY spike records. The P values are for equal means between CTR and IMY groups estimated by Student’s t-test or Mann-Whitney test (*). J_Ca_ – calcium current density; TTP - time to peak amplitude; FDHM – full duration at half-maximum; V_1/2_ - half activation potential; V_r_ - reversal potential; CRS density – the number of calcium release sites per sarcomere; Amp - maximal amplitude, Lat - latency. J_Ca_ was determined as the peak I_Ca_ divided by membrane capacitance; V_1/2_ and V_r_ were determined from of I_Ca_-V curves; I_Ca_ parameters Amp, TTP, and FDHM were determined from calcium currents at 0 mV. The parameters Amp, Lat, TTP, and FDHM of calcium spikes and of integral CRFs were determined from the respective best-fit curves.

### Calcium release changes in IMY myocardia

Calcium currents, calcium spikes, and integral calcium release fluxes were assessed in parallel in isolated cardiac myocytes of both experimental groups with the aim to reveal eventual correlations with the changes observed in the dyadic population. The isolated myocytes of either group showed no abnormalities in optical qualities under the light microscope.

**Figure 3:**
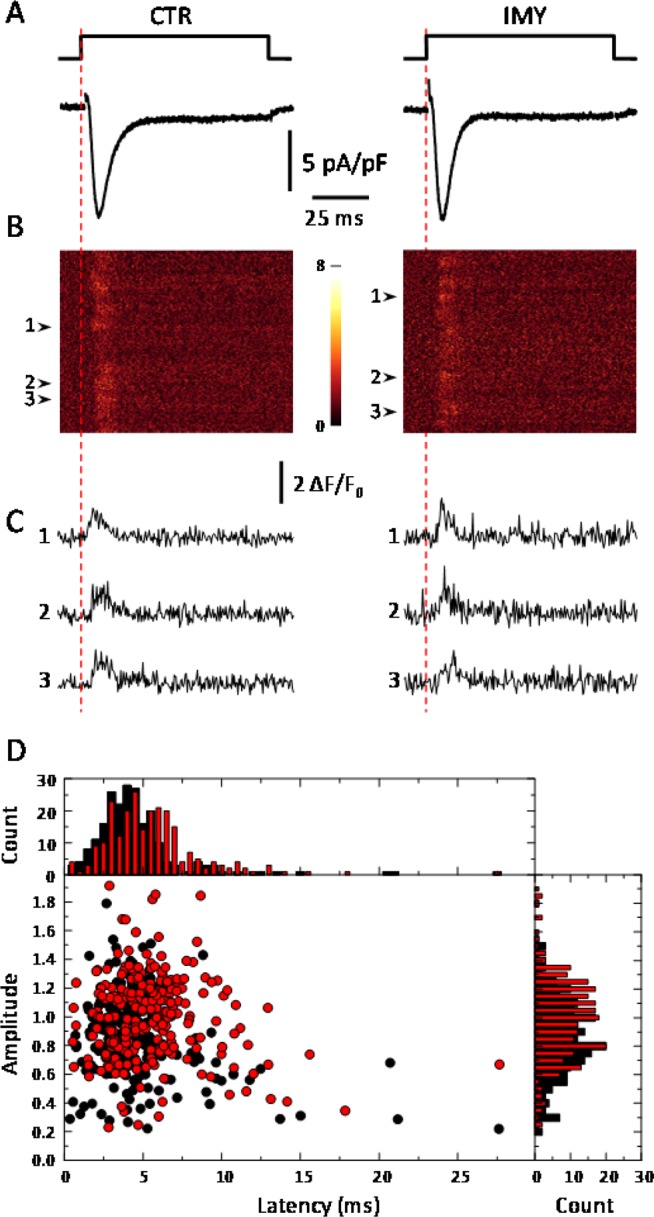
Overview of electrophysiological experiments. A and B illustrate a combined whole-cell patch clamp and confocal fluorescence microscopy experiment in a typical CTR (left) and IMY myocyte (right). (**A**) The voltage stimulus (top traces) and the corresponding calcium current (bottom traces). (**B**) x-t line-scan confocal fluorescence images of calcium spikes recorded simultaneously with calcium currents in A. The numbered arrowheads point to the positions of the release sites generating calcium spikes shown in C. (**C**) The time-fluorescence profiles of the spikes indicated in B. Panels A, B, and C have the same time coordinates. (**D**) The latencies and amplitudes of the recorded calcium spikes from 15 CTR and 17 IMY myocytes. Distribution diagrams of latencies and amplitudes are shown as columns (top and right panels, respectively). Black and red mark CTR and IMY group data, respectively.

Calcium currents recorded at voltage pulses to 0 mV had similar amplitudes and time courses in both experimental groups (Fig. 3A). Neither the density of calcium current, nor the time-to-peak, nor the full-duration at half-maximum changed significantly (Table 2). Similarly, the voltage dependence of calcium current was not changed significantly (Table 2, V_1/__2_ and V_r_). It could be therefore inferred that the trigger function of calcium current in IMY myocytes was preserved at the same level as in control myocytes and that the calcium current itself did not cause the changes in local calcium release observed in these experiments (see below).

Calcium spikes were recorded by a calcium sensitive dye as a local increase in fluorescence intensity triggered by the calcium current (Fig. 3). The positions of calcium spikes defined positions of calcium release sites (CRS). The number of CRSs per sarcomere was not different between the two experimental groups (Table 2, CRS density), and agreed well with the number of dyads per sarcomere determined from electron microscopic images (Table 1). Therefore, any differences in calcium release observed in myocytes of the two experimental groups (Table 2) could not be caused by changes in the density of dyads or calcium release sites.

To overcome the problem of low signal-to-noise ratio in fluorescence records, characteristics of calcium spikes were estimated from fitted curves obtained by fitting Eq. 1 to fluorescence signals^36^. Differences between spikes of the control and the IMY myocytes (Fig. 3B,C) were more apparent when presented in the form of distributions (Fig. 3D). Myocytes of the IMY group produced less calcium spikes with latencies below 5 ms but more calcium spikes with latencies of 6 - 8 ms than the control group, such as if the whole latency distribution shifted toward larger values. The distribution of spike amplitudes of IMY myocytes was also shifted towards larger values over the controls. These shifts gave rise to statistically highly significant differences in the mean latencies and amplitudes between spikes of the two experimental groups (Table 2). The same was valid also for their time to peak and full duration at half maximum. Changes in the mean values of individual parameters were similar, their IMY/CTR ratios ranged between 1.16 and 1.23, indicating that none of spike characteristics was distinctly sensitive or insensitive to the injury.

The variability of calcium spikes produced broad, strongly skewed, and inhomogeneous distributions, in agreement with previous observations^36,37^. Since their probability density distribution functions are unknown, and since changes in calcium spikes were not large enough, decomposition of parameter distributions into underlying components and their correlation with occurrence of different dyad types was not possible. Taken together, however, the clear differences in descriptors of calcium spikes indicate major changes in the function of calcium release sites, and thus in their corresponding dyads, imposed by the injury.

**Figure 4:**
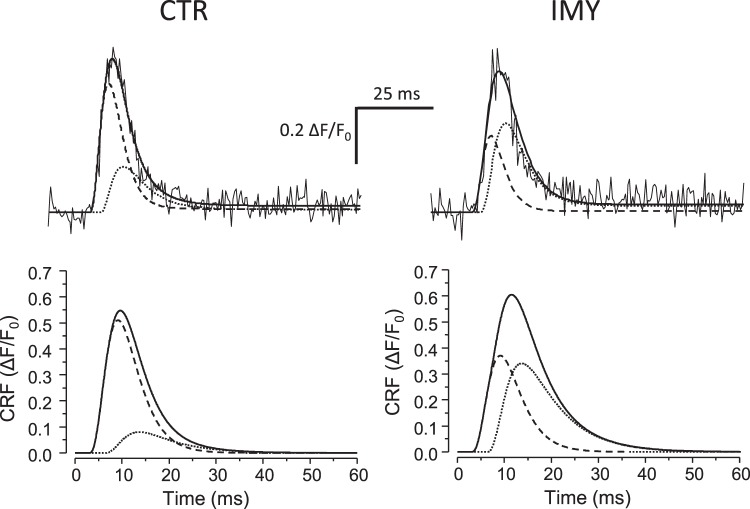
Time courses of integral calcium release fluxes (CRF). Top – Noisy lines - typical time courses of the integral calcium release flux in CTR (left) and IMY (right) myocytes; solid lines - the best-fitted curves of integral CRF (Eq. 2). Dashed and dotted lines – the early and the late components of the best fit curves, respectively. Bottom - The simulated average integral calcium release flux (Eq. 2) with parameters from Table 3. Solid line - the average integral CRF (R = R_1_ + R_2_). Dashed and dotted lines - the average early (R_1_) and late component (R_2_), respectively.

The integral calcium release flux (CRF) was obtained by integration of the fluorescence signal along the scanning laser line at each time point. The amplitude and the time course of the resulting fluorescence signals (Fig. 4, top) varied between sampled areas, reflecting the variability of local calcium release events (Fig. 3D). This integral CRF signals could be well approximated by the phenomenological function (Eq. 1) for triggered transient release flux^36^, and in contrast to the parameter distributions of calcium spikes, the latencies and amplitudes of integral release fluxes were normally distributed. The results of fitting the whole dataset of integral CRFs, summarized in Table 2, reveal that the kinetic characteristics of integral CRFs were substantially changed in myocytes of injured myocardium when compared to controls. Individual characteristics of CRF were affected by the injury to a similar extent as the analogous characteristics of Ca spikes. A closer inspection revealed some differences in the two datasets. The increase in the amplitude of integral CRF was somewhat smaller than that of Ca spikes due to the scatter of spike latencies in the integrated space. The latencies of Ca spikes and of the integral CRF differed due to the fact that the latency of an integral CRF record is determined by the earliest latency of a Ca spike in the integrated space, and not by their mean. The longer TTP and FDHM of integral CRF records could be attributed to the temporal scatter of spikes along the integrated space. To summarize, the calcium release flux in IMY myocytes observed at the local level was of larger amplitude, started with a significantly longer latency, peaked later and lasted substantially longer than in myocytes of the control group.

### Relationships between calcium release flux and dyad structure

The myocardial injury induced changes in the population of both, the dyads and the calcium release sites. The question was whether these structural and functional changes could be related. Essentially the same number of calcium release sites and of dyads per sarcomere, in both control and IMY myocytes (cf. Tables 1 and 2) indicates that the respective datasets were obtained by congruent stochastic sampling of the same experimental space. This justifies the datasets for statistical correlation.

The hypothesis that the two qualitatively distinct structural dyad types give rise to two functionally distinct calcium release sites requested decomposition of the integral calcium release fluxes into independent principal components. To this end, the integral CRF record was described as a sum of two release flux functions (Eq. 2, R_1_ and R_2_) of the same set of parameters that differ in the assumed parameter values. Since the same dyad types exist in both myocyte groups, as we have shown above, the kinetic parameters of the two release functions were estimated as the best collective fit to all integral CRF records, no matter whether obtained in CTR or IMY myocytes. However, since the proportions of the two dyad types vary at different sampled areas, the amplitude parameters of the two flux components were allowed to be optimized independently and specifically for each CRF record. As a result, the integral flux was decomposed into two kinetic components (Fig. 4, top). The two components were of different amplitudes in the CTR and IMY groups (Table 3). The resulting theoretical CRF functions and their components simulated for the two myocyte groups are shown in Fig. 4 (bottom). Notably, the latency, TTP, and FDHM of the two calcium release fluxes were substantially different, what allows to dub them as the early and the late CRF. The two CRF components were of substantial but different amplitude in the two experimental groups (Table 3). This indicates their largely different contribution in CTR and IMY myocytes. The increased contribution of the late CRF component in the IMY myocytes allows associating the source of the late component with the compromised calcium release sites.

**Table 3:** Two-component analysis of integral calcium release fluxes. Amplitudes and fractions are given as mean ± s.e.m. obtained from 15 CTR and 17 IMY traces. Amplitude – amplitudes of the best fit curves to CRF records. Student’s t-test p-values for equal means between the CTR and IMY groups were P = 0.01 for the early components, and P = 2.2E-5 for the late components. Fraction – the amplitude of one component of release flux divided by the sum of amplitudes of early and late components; calculated for each best fit curve. Student’s t-test p-values for equal means between the CTR and IMY groups were P = 2.7E-5 for the both, early and late components. Lat – latencies of the best fit the early and late components estimated for all 32 (CTR plus IMY) curves collectively, given as mean ± standard error of the fit.TTP and FDHM were determined from the simulated curves of the early and late components visualized in Fig. 4 (bottom).The same experimental data set as in Table 2.

	Early component		Late component	
	CTR	IMY	CTR	IMY
Amplitude (ΔF/F0)	0.51 ± 0.03	0.37 ± 0.04	0.08 ± 0.01	0.34 ± 0.04
Fraction	0.85 ± 0.03	0.52 ± 0.05	0.15 ± 0.03	0.48 ± 0.05
Lat (ms)	2.97 ± 0.14		6.29 ± 0.16	
TTP (ms)	6.19		8.32	
FDHM (ms)	8.32		12.21	

### Correlation of morphometric and electrophysiological data

**Figure 5:**
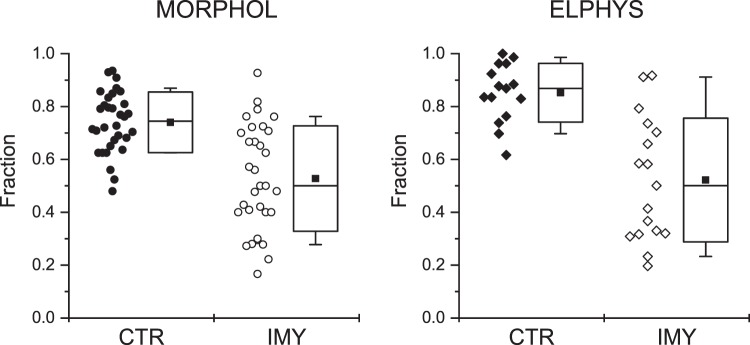
Fractions of the unperturbed elements of calcium release in cardiac myocytes. MORPHOL: fractions of compact dyads estimated by morphometry from electron microscopic images (Table 1). ELPHYS: fractions of the early CRF components estimated by fitting records of integral fluorescence signals (Fig. 4). CTR - control myocardium; IMY - injured myocardium. All collected data are shown. Box plots show the 25%, 50% and 75% percentiles; whiskers show 10% and 90% percentile. Solid squares denote the means.

The correlation between structural and functional data (Fig. 5) was examined using two-way ANOVA^38^. The two considered treatment factors were the method of measurement ‘Method’ and the experimental intervention ‘Intervention’. The factor ‘Method’ included two levels - the morphometric (Morphol) and the electrophysiological (Elphys) methods of measurement. The factor ‘Intervention’ also included two levels - the control myocardium (CTR) and the injured myocardium (IMY). The common examined variable was the fraction of unperturbed elements of calcium release, namely, the fraction of compact dyads in the Morphol level and the fraction of early CRF components in the Elphys level, since these dominate in control myocytes. The estimated values of the variables are given in Fig. 5. No outliers were identified. The data did not show significant differences from normality (Shapiro-Wilk test: P = 0.79, 0.37, 0.54, 0.22 for the CTR-Morphol, IMY-Morphol, CTR-Elphys and IMY-Elphys groups, respectively). The both data sets of CTR groups had significantly smaller variances than that of the IMY groups (0.013 and 0.040, P = 0.003 for the Morphol level; 0.012 and 0.055, P = 0.008 for the Elphys level, F-test), indicating that the variance of IMY groups arose from a biological reason and not from incorrect sampling.

Two-way ANOVA test revealed that the effect of the treatment factor ‘Intervention’ (IMY *vs*. CTR) on the fraction of unperturbed elements was highly significant (P < 1 × 10^−10^) while that of the treatment factor ‘Method’ (Morphol *vs*. Elphys) was not significant (P > 0.1). This means that the overall fraction of unperturbed elements of calcium release was significantly reduced by the myocardial injury. Moreover, it indicates that the method of observation (morphometric vs. electrophysiological) did not affect the observed fractions of unperturbed elements of calcium release; in other words, the error of sampling was similar for both methodological approaches.

Analysis of simple effects^38^ showed that in control myocytes, the fraction of unperturbed elements of calcium release estimated by morphometry (0.74 ± 0.04; Table 1, Compact/All dyads) and that estimated by electrophysiology (0.85 ± 0.03; Table 3, Amp fraction) were significantly different (P < 0.005, Mann-Whitney test).

To summarize, the two-way ANOVA tests of data in Fig. 5 revealed that compact dyads and early CRFs can be considered equivalent, and in both animal groups do represent the same unperturbed elements of calcium release. The same is valid for the equivalence between loose dyads and late CRFs. Moreover, the adaptation of myocardium to injury caused a clear shift in the population of calcium release elements towards the perturbed forms.

## Discussion

It was previously anticipated that the structure of dyads plays an important role in cardiac myocytes, especially in the efficiency of calcium release during excitation-contraction coupling^11,16,27,39–42^, but experimental evidence was lacking. To disclose the relationship between the structure and the function of dyads, we designed a specific methodology. It was based on collection of structural data by methods of electron microscopy and of functional data by methods of electrophysiology. Collected data of both the structural and the functional type were categorized, each into two subtypes, and the four categories were examined by two-way ANOVA.

We found that the compact dyads and the early component of calcium release flux represent equivalent entities, and that the complementary pair, the loose dyads and the late component of CRF, represent other equivalent entities (Table 3; Fig. 5). It can be assumed that subtler distinction of traits, for instance based on morphometry of dyads, could reveal more detailed correlations. Very likely this may not be experimentally resolved, because the sub-microscopic variability of molecular assemblies of small number of proteins, such as RyRs in dyads or calcium release sites, would likely blur the observations of their local stochastic operation. The small-scale structural dynamics could contribute to the observed broad distribution of spike parameters (Fig. 3D).

It could be that the high statistical correlation between the structural and functional traits was only a chance observation in these experiments, and that the real reason behind the changed calcium release was not in the changed structure of CRSs but in pharmacological modification of RyRs or their regulatory mechanisms enforced by the isoproterenol treatment. Our data do not support this alternative interpretation, since the calcium current through DHPR channels, which are sensitive to pharmacological effects of isoproterenol^43^ and which are co-localized with RyRs in dyads^12^, was not changed at all (Table 2).

We found that the quality of calcium release sites varied broadly even in control myocytes of rats housed under standard conditions, although the compact dyads and early calcium releases dominated in this group (Table 1). In this context it is of interest that in the injured myocardium the same types of CRSs were observed as in the control myocardium; just the population of CRSs was shifted towards the perturbed forms. This finding indicates that perturbations of calcium release related to the dyad structure have arisen from biological mechanisms inherent to cardiac myocytes, and that these mechanisms activate in parallel to the disbalance in myocardial function. This mechanism might play a role in adaptation of myocardium to physiological and pathological conditions of growth, lifestyle, pregnancy, stress, hypertrophy, etc. It would be of interest to know whether the state of calcium release sites, as observed on day 15 after the experimental injury, is a transient state that would eventually develop in time toward dominance of unperturbed CRSs similar to control myocytes, or whether by developing toward increased prevalence of perturbed CRS it would become a part of the heart pathology.

The changes in calcium spikes (Table 2) revealed two faces of the same coin. The larger average amplitude of calcium spikes, that is, of the calcium release flux at an average CRS, points to possibly potentiated contractility of working myocytes of IMY hearts. On the other hand, the slower kinetics of calcium release flux may cause problems to hearts beating at higher rates. The presented experiments do not allow explanation of molecular mechanisms behind the changes in CRS function, since dynamics of calcium spikes reflects group behaviour of RyR channels and the distribution of calcium ions at the terminal cisternae, lumen of sarcoplasmic reticulum, and cytosol. Nevertheless, at each time moment the amplitude of calcium release flux is the product of the number of open RyR channels and of the concentration gradient of calcium ions^37,44^. According to the kinetic description of local CRF (Eq. 1), the momentary amplitude of release depends on the maximal achievable amplitude, proportional to the number of RyRs in a CRS. The individual RyRs are switched on by an activation process, and switched off by a termination process. These processes depend on local calcium concentration in a complex way, including calcium diffusion and binding. If the calcium concentration was similar at all release sites in a myocyte, then the diffusion and binding of calcium ions would depend on the geometrical disposition of individual CRSs. When the construction is compact, the space factors are optimal. When the construction is loose, the space factors become suboptimal. Electron microscopic images indicate that possible structural factors could be the state of calsequestrin and the dimensions of terminal cisternae. According to this study, the condensed calsequestrin and narrow cisternae characterizing the compact dyads associate with the early component of CRF.

The differences between the integral CRF of CTR and IMY spikes largely paralleled the difference in characteristics of calcium spikes of CTR and IMY myocytes (Table 2). The integral calcium release flux started with a significantly longer latency, peaked later and lasted substantially longer in IMY than in control myocytes. Such changes point to reduced DHPR-RyR coupling fidelity^8,39^ and prolonged recruitment of dyads^8^. The slowing of integral calcium release flux in IMY myocytes can be also partially assigned to the less synchronized activation of the involved dyads^11,33,34^.

In control myocytes, the mean fractions of unperturbed elements (the early CRF and the compact dyad) were somewhat different, while their dispersions were small (Fig. 5, solid symbols). It was not the case in IMY myocytes, where the mean fractions of unperturbed elements were similar, while their dispersions were large (Fig. 5, open symbols). This could mean that some anomalies in dyadic structures, leading to their classification as loose dyads, might not have a measurable impact on the local calcium release flux. This small disproportion could be understood in line with recent understanding of the dyad as a variable cluster of ryanodine receptors^20,21^ that functions on stochastic grounds^16,37^. In other words, the response of a single dyad with a partially perturbed structure might not respond to all stimuli by late calcium spikes, especially if the dyad contained large clusters of RyRs. The complementary argument that neither the perfect dyads always respond perfectly should be valid as well; therefore, the above disproportion in data measured in the CTR myocyte group should be considered as the result of larger disproportions in stochastic sampling and stochastic gating in the control than in the injured myocardium. Alternatively, the relationship between fractional contents of dyads and fractional contents of release flux might not be linear.

Technically, the decisions about classification of a dyad according to its structure were in fact made on the part of the dyad in the image. The dyad size is not a quantitatively well-defined entity, since it is not an organelle with exactly defined borders, and its size in 3D reconstructions varies broadly^18–21^. Thus, depending on the dyad size and its orientation in the section, the volume of the dyad sampled in this study could represent about 10–50% of its real volume. From statistical point of view, the observed fraction of a dyad in the section was a representative, albeit variable fraction of the whole dyad. The uncertainty associated with sampling and classification of dyads necessitated collection of a large dataset of dyad images. It should be also considered that a large dyad may not be structurally homogenous; one part may be compact while the other loose, as if it was composed of several CRSs. Therefore, a population of two structural dyad types could be also thought of as a population of dyadic subdomains of the two types. Moreover, according to recent evidence^19,20^, the dyad may house several RyR clusters of variable size, which may function independently. Therefore, the part of the dyad in the section represents a substantial fraction of the RyR cluster, which functions as a calcium release site, observed functionally as the source of calcium spark. Therefore, the error of association of a dyad image with the calcium release site should not depreciate statistical comparisons.

To summarize, we have found that cardiac ventricular myocytes of rats contain two structural types of dyads - the compact and the loose. In healthy rats, the compact dyads dominate. Myocardial injury imposed by isoproterenol treatment decreased the fraction of compact dyads. We have also found that calcium release sites respond to electrical stimulation by calcium release flux, consisting of two components - the early and the late. Myocardial injury decreased the fraction of early calcium release flux component. Statistical testing confirmed a strong correlation between the fraction of compact dyads and the fraction of early calcium release flux. Remodelling of dyads in response to myocardial injury led to corresponding changes in the function of calcium release sites. These findings provide evidence on the relationship between the structural quality of dyads and functional quality of calcium release sites.

## Materials and Methods

### Animal experiments

The study was approved by the Ethics Committee of the Institute of Molecular Physiology and Genetics, Slovak Academy of Sciences and by the State Veterinary and Food Administration of the Slovak Republic (protocols No: Ro-2222/06-221 and Ro-2445/09-221) and conformed to the European directive 2010/63/EU and to Act No. 377/2012 of the Government of the Slovak Republic.

Male rats (Wistar Han, 240–310 g, age 12–16 weeks, Breeding Farm Dobra Voda, reg. No. SK CH 24011, Slovak Republic) fed *ad libitum* were used in the study. A single high dose of isoproterenol (150 mg/kg, Fluka, Steinheim, Germany) in vehicle (0.05% ascorbic acid in 0.9% NaCl, Centralchem, Bratislava, Slovak Republic) was applied subcutaneously to induce myocardial injury (IMY) in rats as described by Grimm *et al*.^45^. Rats of control (CTR) group were injected with the vehicle only. Hearts of both animal groups were used for experiments on day 15 after treatment. Altogether 43 animals were used for the study (17 animals for functional, biometric and electrocardiographic (ECG) studies; 8 animals for electron microscopy studies; 18 animals for patch-clamp and confocal microscopy studies).

Rats were sacrificed by heart dissection under appropriate anaesthesia, as described for specific experiments. Deep anaesthesia was verified by cessation of the corneal and the paw withdrawal reflexes. Excised hearts were mounted on the Langendorff perfusion setup either for *ex vivo* recording, or for isolation of myocytes, or for whole heart fixation.

### Solutions

Krebs-Henseleit (K-H) solution (in mmol/l): 118 NaCl, 4.7 KCl, 1.66 MgCl_2_, 2 CaCl_2_, 1.18 KH_2_PO_4_, 15 NaHCO_3_, 11 glucose, pH 7.35, saturated with 95% O_2_ and 5% CO_2_.

Tyrode solution (in mmol/l): 135 NaCl, 5.4 KCl, 5 MgCl_2_, 1 CaCl_2_, 0.33 NaH_2_PO_4_, 10 HEPES, pH 7.3.

Ca-free Tyrode solution (in mmol/l): 135 NaCl, 5.4 KCl, 5 MgCl_2_, 0.02 CaCl_2_, 0.33 NaH_2_PO_4_, 10 HEPES, pH 7.3.

Enzyme solution (in mmol/l): 135 NaCl, 5.4 KCl, 5 MgCl_2_, 0.02 CaCl_2_, 0.33 NaH_2_PO_4_, 10 HEPES, 0.19 U ml^−1^ Liberase Blendzyme 3 (Roche Diagnostics, Basel, Switzerland), pH 7.3.

Storage solution (in mmol/l): 106 CH_3_SO_3_H, 106 KOH, 3.9 KCl, 2.4 MgSO_4_, 8 K_2_HPO_4_, 1 ethylene glycol-bis(2-aminoethylether)-N,N,N′,N′-tetraacetic acid (EGTA), 22 taurine, 22 glucose, pH 7.3.

Cacodylate buffer (in mmol/l): 150 Na cacodylate, 2 CaCl_2_, pH 7.3.

External solution (in mmol/l): 135 NaCl, 5.4 CsCl, 10 HEPES, 5 MgCl_2_, 0.33 NaH_2_PO_4_, 1 CaCl_2_, 0.01 IBMX, 0.02 TTX, pH 7.3.

Internal solution (in mmol/l): 135 CsCH_3_SO_3_, 10 CsCl, 10 HEPES, 2 EGTA, 2 CaEGTA, 3 MgSO_4_, 3 ATPNa_2_, 1 Oregon Green BAPTA-5N (Thermo Fisher Scientific, Brno, Czech Republic), 0.05 cAMP, pH 7.3; 100 nM free [Ca^2+^].

Chemicals were from Sigma-Aldrich (USA), if not stated otherwise.

### Ventricular electrocardiography and morphology

ECG recording was performed on conscious standing rats after five days handling. Standard 4-lead records were made through limbs in a restraint cage with plate electrodes mounted at the base^46^ and connected to the recording and analysis system (EKG Praktik Veterinary, Seiva, Prague, Czech Republic).

The contractile performance of left ventricles was assessed *ex vivo* in spontaneously beating hearts. For these experiments, rats were anesthetized with Thiopental (45 mg/kg, 5% solution i.p.; Biochemie Gmbh, Kundl, Austria). The hearts were rapidly excised into ice-cold K-H solution and retrogradely perfused in the Langendorff perfusion setup equipped with a peristaltic pump (PLP 33, Heidolph, Schwabach, Germany) in constant pressure mode (80 mm Hg). Before recording, the hearts were stabilised by perfusion with oxygenated K-H solution at 37 °C for 30 min^31^. The left ventricular end-diastolic pressure was monitored by a balloon (No. 5, HSE, Freiburg, Germany) and a pressure transducer (MLT844, ADInstruments, Spechbach, Germany). The basal pressure was set to 10 mm Hg.

Electrical activity of the left ventricular free wall of perfused hearts was recorded by impaled needle electrodes (MLA1213, ADInstruments) connected to amplifier and controller system (FE136 Animal Bio Amp, Powerlab 8/30, ADInstruments), and analysed by Chart 5 (ADInstruments). Coronary flow was measured by timed collection of the coronary effluent into a calibrated column. After completion, the hearts were placed in ice-cold K-H solution to stop the heartbeat and to collect biometric data^47^.

### Electron microscopy and morphometry

The structure of cardiac myocytes was assessed in heart tissue processed for electron microscopy. Hearts were excised under pentobarbital anaesthesia (sodium pentobarbital, 100 mg kg^−1^ i.p.), mounted on a Langendorff setup, perfused with oxygenated Tyrode solution at 37 °C for 5 minutes to remove blood, then for 5 minutes with calcium-free Tyrode solution to relax the heart, and finally for 15 minutes with 2% glutaraldehyde in cacodylate buffer to pre-fix the heart. Tissue samples of about 1 mm^3^ were dissected from a site near the base of the heart, in the case of IMY animals located >5 mm from the lesion that was always located near the apex. Samples were fixed for additional 45 min with 2% glutaraldehyde in cacodylate buffer, postfixed with 1% osmium tetroxide in cacodylate buffer for 30 min, contrasted with 1% uranyl acetate in H_2_O, dehydrated in a series of alcohol/water mixtures and embedded through dry acetone in Durcupan (Fluka, Geneva, Switzerland). The samples were dissected from the endocardial layer of the upper part of the left ventricular free wall, since this region was always outside of the unperfused ischemic zone in IMY hearts. Four sample blocks were prepared from each of four CTR and four IMY group hearts. The tissue in the block was oriented so that the sectioning plane was parallel to the longitudinal axis of myocytes.

Random longitudinal ultrathin sections (58 nm) were cut from each sample block with Power-Tome MT-XL (RMC/Sorvall, Tucson, USA) and contrasted on grid with lead citrate. The sections were viewed using a transmission electron microscope (JEM 1200, JEOL, Tokyo, Japan) and images were recorded using a CCD camera (Dual Vision 300 W, Gatan, Pleasanton, USA). From each section, a random set of images was taken at 15 000× magnification.

Dyads were characterized using an analysis window of 1 µm in width, sliding parallel to myocyte’s longitudinal axis at a distance of 3–5 µm from the plasma membrane. Two randomly selected myocytes per block were analysed. The dyads were classified (see section *Structure of dyads varies in cardiac myocytes*) and their occurrence was counted over 30 sarcomeres per myocyte. The size and position of the analysis window was designed to make morphometric measurements compatible with measurements of calcium signals by laser scanning fluorescent confocal microscopy. The dyads in the images were classified by three evaluators who were not informed about the sample source (CTR vs. IMY). The number of dyads of individual type in each myocyte was taken as the mean of individual evaluations. The overall difference between classifications by individual evaluators was less than 5%.

### Myocyte isolation

Before chest opening, rats were heparinized (5000 U kg^−1^ i.p.) and deeply anesthetized with sodium pentobarbital (100 mg kg^−1^ i.p.). Excised hearts were perfused in retrograde using a Langendorff setup. Blood was washed out by 5-minute perfusion with Tyrode solution, followed by 5-minute perfusion with Ca^2+^-free Tyrode solution, and 5 minutes with the enzyme solution, all oxygenated at 37 °C. The digested tissue was transferred to the storage solution at room temperature, trimmed to small chunks, triturated, and filtered through a nylon mesh to remove the undigested tissue. Myocytes were kept in the storage solution at room temperature of about 23 °C.

### Patch-clamp and confocal microscopy

Isolated cardiac myocytes were patch-clamped using Axopatch 200B amplifier, Digidata 1320 A A/D converter, and pClamp Ver. 9 (Molecular Devices, Sunnyvale, USA) in the whole-cell configuration to stimulate the cell, to estimate the cell capacitance, and to record simultaneously the calcium current, I_Ca_, and calcium spikes^36^. Patch-clamped myocytes were held at the resting potential of -50 mV. Cell capacitance was measured immediately after obtaining the whole-cell recording configuration. Calcium currents and calcium spikes were activated once per 30 s by 70-ms voltage pulses from -50 to 0 mV during 5 to 10-minute interval after the whole-cell configuration was established. Voltage stimuli were synchronized with image acquisition. In some cells, calcium current – voltage curves were determined by recording calcium currents at a range of voltage pulses from -40 to +50 mV in 10 mV increments, and plotting the peak I_Ca_ amplitude against membrane voltage^36^.

Leica TCS SP2 AOBS confocal microscope with PlanApochromat 63×/1.32 NA oil immersion objective (Leica Microsystems, Heidelberg, Germany) was used to record fluorescence signals as described previously^36^. Calcium release flux was measured using 1 mM fluorescent calcium indicator OG-5N and 4 mM EGTA, adjusted to 90 nM free [Ca^2+^], and dialyzed into the cell through the patch pipette. Confocal aperture set to 2.5 Airy provided optical sections of about 980 nm thick. The focus was set at 3–5 µm from the surface of the myocyte. The cell was scanned along a 60-μm line parallel to the longitudinal axis at 2000 Hz bi-directionally. The resulting x-t images of 116 nm and 0.5 ms per pixel allowed resolving calcium spikes emerging at individual release sites.

### Analysis of calcium release flux

The background fluorescence measured in a region not containing the cell was subtracted from the whole x-t image. The resulting image was divided by the average fluorescence intensity of the cell region scanned before the onset of voltage stimulus. The final *ΔF*∕*F*_0_ image was averaged on the x coordinate. The resulting time course of fluorescence intensity was used as a measure of the integral calcium release flux (CRF)^36,48^.

Parameters of individual calcium spikes as well as of local integral CRFs were determined by fitting their time course in SpikeAnalyzer software^9,36^ with the function:

1 $$\begin{array}{c}{\rm{CRF}}(t)=R(t,\,{F}_{M},\,\alpha ,\,{t}_{0},\,{\tau }_{A},\,{\tau }_{T})=\frac{{{\rm{F}}}_{{\rm{M}}}}{({\tau }_{A}-3{\tau }_{I})\,({\tau }_{A}-2{\tau }_{T})\,({\tau }_{A}-{\tau }_{T})}\cdot \\ +\frac{1}{3}{{\rm{e}}}^{-\frac{3(t-{{\rm{t}}}_{0})}{{{\rm{\tau }}}_{{\rm{A}}}}}{{\rm{\tau }}}_{{\rm{A}}}({{{\rm{\tau }}}_{{\rm{A}}}}^{2}(57-{10{\rm{\alpha }}{\rm{\tau }}}_{{\rm{A}}})-{3{\rm{\tau }}}_{{\rm{A}}}(84-{13{\rm{\alpha }}{\rm{\tau }}}_{{\rm{A}}}){{\rm{\tau }}}_{{\rm{T}}}+(249-{29{\rm{\alpha }}{\rm{\tau }}}_{{\rm{A}}}){{{\rm{\tau }}}_{{\rm{T}}}}^{2})+\\ +\frac{{{\rm{18e}}}^{-\left(\frac{1}{{{\rm{\tau }}}_{{\rm{A}}}}+\frac{1}{{{\rm{\tau }}}_{{\rm{T}}}}\right)(t-{{\rm{t}}}_{0})}{{{\rm{\tau }}}_{{\rm{T}}}}^{3}({{\rm{\tau }}}_{{\rm{A}}}+{{\rm{\tau }}}_{{\rm{T}}}-{{\rm{\alpha }}{\rm{\tau }}}_{{\rm{A}}}{{\rm{\tau }}}_{{\rm{T}}})}{{{\rm{\tau }}}_{{\rm{A}}}+{{\rm{\tau }}}_{{\rm{I}}}}-\frac{{{\rm{18e}}}^{-\left(\frac{2}{{{\rm{\tau }}}_{{\rm{A}}}}+\frac{1}{{{\rm{\tau }}}_{{\rm{T}}}}\right)(t-{{\rm{t}}}_{0})}{{{\rm{\tau }}}_{{\rm{T}}}}^{3}({{\rm{\tau }}}_{{\rm{A}}}+(2-{{\rm{\alpha }}{\rm{\tau }}}_{{\rm{A}}}){{\rm{\tau }}}_{{\rm{T}}})}{{{\rm{\tau }}}_{{\rm{A}}}+{2{\rm{\tau }}}_{{\rm{I}}}}+\\ +\frac{{\rm{\alpha }}({{\rm{\tau }}}_{{\rm{A}}}-{3{\rm{\tau }}}_{{\rm{T}}})\,({{\rm{\tau }}}_{{\rm{A}}}-{2{\rm{\tau }}}_{{\rm{T}}})\,({{\rm{\tau }}}_{{\rm{A}}}-{{\rm{\tau }}}_{{\rm{T}}})\,({{37{\rm{\tau }}}_{{\rm{A}}}}^{4}+{{252{\rm{\tau }}}_{{\rm{A}}}}^{3}{{\rm{\tau }}}_{{\rm{T}}}+{{605{\rm{\tau }}}_{{\rm{A}}}}^{2}{{{\rm{\tau }}}_{{\rm{T}}}}^{2}+{660{\rm{\tau }}}_{{\rm{A}}}{{{\rm{\tau }}}_{{\rm{T}}}}^{3}+{{360{\rm{\tau }}}_{{\rm{T}}}}^{4})}{60\,({{\rm{\tau }}}_{{\rm{A}}}+{{\rm{\tau }}}_{{\rm{T}}})\,({{\rm{\tau }}}_{{\rm{A}}}+{2{\rm{\tau }}}_{{\rm{T}}})\,({{\rm{\tau }}}_{{\rm{A}}}+{3{\rm{\tau }}}_{{\rm{T}}})}+\\ (-\frac{3}{2}{e}^{-\frac{2(t-{{\rm{t}}}_{0})}{{{\rm{\tau }}}_{{\rm{A}}}}}{{\rm{\tau }}}_{{\rm{A}}}(2-{{\rm{\alpha }}{\rm{\tau }}}_{{\rm{A}}})\,({4{\rm{\tau }}}_{{\rm{A}}}-{7{\rm{\tau }}}_{{\rm{T}}})\,({{\rm{\tau }}}_{{\rm{A}}}-{3{\rm{\tau }}}_{{\rm{T}}})+3{e}^{-\frac{(t-{{\rm{t}}}_{0})}{{{\rm{\tau }}}_{{\rm{A}}}}}{{\rm{\tau }}}_{{\rm{A}}}({{\rm{\tau }}}_{{\rm{A}}}-{3{\rm{\tau }}}_{{\rm{T}}})\,({{\rm{\tau }}}_{{\rm{A}}}-{2{\rm{\tau }}}_{{\rm{T}}})+\\ \,\frac{3}{5}{{\rm{e}}}^{-\frac{5(t-{{\rm{t}}}_{0})}{{{\rm{\tau }}}_{{\rm{A}}}}}{{\rm{\tau }}}_{{\rm{A}}}(5-{{\rm{\alpha }}{\rm{\tau }}}_{{\rm{A}}})\,({2{\rm{\tau }}}_{{\rm{A}}}-{5{\rm{\tau }}}_{{\rm{T}}})\,({{\rm{\tau }}}_{{\rm{A}}}-{{\rm{\tau }}}_{{\rm{T}}})-\frac{1}{6}{{\rm{e}}}^{-\frac{6(t-{{\rm{t}}}_{0})}{{{\rm{\tau }}}_{{\rm{A}}}}}{{\rm{\tau }}}_{{\rm{A}}}(6-{{\rm{\alpha }}{\rm{\tau }}}_{{\rm{A}}})\,({{\rm{\tau }}}_{{\rm{A}}}-{2{\rm{\tau }}}_{{\rm{T}}})\,({{\rm{\tau }}}_{{\rm{A}}}-{{\rm{\tau }}}_{{\rm{T}}})-\\ {{\rm{6e}}}^{-\frac{t-{{\rm{t}}}_{0}}{{{\rm{\tau }}}_{{\rm{T}}}}}{{{\rm{\tau }}}_{{\rm{I}}}}^{3}(1-{{\rm{\alpha }}{\rm{\tau }}}_{{\rm{T}}})-\frac{3}{4}{{\rm{e}}}^{-\frac{4(t-{{\rm{t}}}_{0})}{{{\rm{\tau }}}_{{\rm{A}}}}}{{\rm{\tau }}}_{{\rm{A}}}(4-{{\rm{\alpha }}{\rm{\tau }}}_{{\rm{A}}})\,({{5{\rm{\tau }}}_{{\rm{A}}}}^{2}+{20{\rm{\tau }}}_{{\rm{A}}}{{\rm{\tau }}}_{{\rm{T}}}+{{17{\rm{\tau }}}_{{\rm{T}}}}^{2})+\\ +{{\rm{6e}}}^{-\left(\frac{3}{{{\rm{\tau }}}_{{\rm{A}}}}+\frac{1}{{{\rm{\tau }}}_{{\rm{T}}}}\right)(t-{{\rm{t}}}_{0})}\frac{{{{\rm{\tau }}}_{{\rm{T}}}}^{3}({{\rm{\tau }}}_{{\rm{A}}}+(3-{{\rm{\alpha }}{\rm{\tau }}}_{{\rm{A}}}){{\rm{\tau }}}_{{\rm{T}}})}{{{\rm{\tau }}}_{{\rm{A}}}+{3{\rm{\tau }}}_{{\rm{T}}}}-{{\rm{6e}}}^{-\frac{2(t-{{\rm{t}}}_{0})}{{{\rm{\tau }}}_{{\rm{A}}}}}{{{\rm{\alpha }}{\rm{\tau }}}_{{\rm{A}}}}^{2}({{\rm{\tau }}}_{{\rm{A}}}-{3{\rm{\tau }}}_{{\rm{T}}})\,({{\rm{\tau }}}_{{\rm{A}}}-{2{\rm{\tau }}}_{{\rm{T}}})\cosh \left(\frac{t-{{\rm{t}}}_{0}}{{{\rm{\tau }}}_{{\rm{A}}}}\right)),\end{array}$$

where *R* is the function describing the time course of release flux; *t* is the time elapsed from the start of the voltage stimulus; t_0_ is the latency of the calcium spike; F_M_ is the maximal normalized fluorescence increase in the absence of release termination; α is a proportionality factor; and τ_A_ and τ_T_ are the time constants of activation and termination of release flux, respectively^36^.

The fitted parameters were used to simulate the best-fit trace, from which descriptors of the calcium release flux were estimated^36,48^, namely: the amplitude of maximal fluorescence increase (A, the peak amplitude) as a measure of calcium release flux intensity; the time to peak of release flux (TtP), as a measure of the rate of calcium release activation (the time of the peak amplitude minus the latency determined directly by fitting); and the full duration at half maximum (FDHM), as a measure of calcium release duration.

### Decomposition of calcium release flux into two components

Integral CRF was interpreted by decomposition into two components, which were assumed to represent calcium flux produced by the two types of CRSs (dyads) supposedly differing in the quality of calcium release. To this end, the line integrals of fluorescence traces of all CTR as well as of IMY myocytes were fitted as a single dataset with the sum of two integral CRF functions with shared parameter values:

2 $${\rm{CRF}}(t)={R}_{1}(t,\,{F}_{{\rm{M}}1,i},\,{\alpha }_{1},\,{t}_{01},\,{\tau }_{{\rm{A}}1},\,{\tau }_{{\rm{T}}1})+{R}_{2}(t,\,{F}_{{\rm{M}}2,i},\,{\alpha }_{2},\,{t}_{02},\,{\tau }_{{\rm{A}}2},\,{\tau }_{{\rm{T}}2}),$$

where indexes 1 and 2 correspond to the two release flux components, respectively, and the index *i* denotes individual CRF traces. In other words, the values of the parameters characterizing the kinetics of the two release flux components (t_01_, t_02_, τ_A1_, τ_A2_, τ_T1_, τ_T2_, α_1_ and α_2_) were optimized collectively using the shared parameters setting, while the amplitude parameters F_M1_ and F_M2_ were specific for each trace. To avoid errors that could arise from interdependence between parameters (the problem of many parameters), the characteristics describing the time course of the recorded calcium release flux for each release flux component, that is, the amplitude, the time-to-peak, and the full-duration-at-half-maximum were determined from the best fit curves and used for comparison of experimental groups (see Table 3).

### Calcium release site density and distribution

The area of an x-t image corresponding to 10 ms around the peak of calcium release flux was averaged along the temporal axis. The resulting line fluorescence intensity profiles contained peaks at positions of calcium release sites. The number of peaks per scanned line was counted and related to the number of sarcomeres visible in the transmitted light channel imaging the same region.

### Statistics

All recorded data were analysed using SpikeAnalyzer software^9,36^ or Origin (Ver. 8 SR6 OriginLab Corporation, Northampton, USA). Data are presented as the mean with the standard error, or as the best fit with the standard error of the fit. Statistical significance was tested using Origin. The P values were calculated by the t-test for two-tailed distributions. The t-test statistics was adjusted for equality/inequality of variance when appropriate. Equality of variances was tested using Levene’s test. Significance of differences between variances was tested using the F-test. Normality of distribution was tested by the Shapiro-Wilk and Anderson-Darling test in the program Mathematica (Ver. 11, Wolfram Research, Champaign, IL, USA). Latency and amplitude of calcium spikes and occurrence of individual dyad classes, which did not follow normal distribution, were compared using Mann-Whitney test. Two-way ANOVA tests were performed in STATISTICA (Ver. 11, Dell Statistica, Tulsa, USA).

## Supplementary information

Supplemental Data.
