# Supplementary material for: Structural variability of dyads relates to calcium release in rat ventricular myocytes
Source: Sci Rep. 2020 May 15;10:8076. doi: 10.1038/s41598-020-64840-5 (PMC7229197; doi:10.1038/s41598-020-64840-5)
Supplement: Supplementary file 1 — Supplemental Data. [file 41598_2020_64840_MOESM1_ESM.pdf]

SUPPLEMENTAL DATA

Structural variability of dyads relates to calcium release in rat ventricular myocytes

Marta Novotová[a,b], Alexandra Zahradníková[a,b], jr., Zuzana Nichtová[a], Radoslav Kováč[c], Eva Kráľová[c], Tatiana Stankovičová[c], Alexandra Zahradníková[a,b], and Ivan Zahradník*[a,b]

[a] *Department of Cellular Cardiology, Institute of Experimental Endocrinology, Biomedical Research Center, University Science Park for Biomedicine, Slovak Academy of Sciences, Bratislava, Slovakia,*

[b] *Department of Muscle Cell Research, Institute of Molecular Physiology and Genetics, Centre of Biosciences, Slovak Academy of Sciences, Bratislava, Slovakia, and*

[c] *Department of Pharmacology and Toxicology, Faculty of Pharmacy, Comenius University, Bratislava, Slovakia.*

SUPPLEMENTAL RESULTS

The experimental model of myocardial injury (IMY) was based on a single subcutaneous application of high dose of isoproterenol which causes irreversible infarction-like ischemic damage in the apex [1,2]. The role of IMY model in this study was to induce remodelling of myocytes by increased load on working myocardium. The effect of isoproterenol intervention was verified at the whole heart level. Two weeks after isoproterenol administration, the IMI hearts displayed clear signs of myocardial injury in agreement with previously published data [1,3-5]. This included well visible pale plaques near the heart apex Biometric data indicated a tendency toward development of myocardial hypertrophy (Table S1). The heart weight to body weight index was larger by 25% relative to the control rats. The mild increase in thickness of left ventricles did not reach statistical significance (Table S1). The presence of myocardial injury was supported by *in vivo* and *ex vivo* ECG data, especially by the substantial reduction of R_{II} amplitude and augmented negativity of S_{III} wave (Supplemental Table 1). A substantial increase of the heart rate and prolongation of the QTc interval, both *in vivo* and *ex vivo*, together with the prolonged QRS complex, illustrate a persisting electrical imbalance in IMY hearts. This was underlined by episodes of ventricular fibrillation incurred in three of nine IMY hearts. In spontaneously beating isolated hearts, perfused under Langendorff setup, the systolic pressure

developed by left ventricles was substantially decreased while the diastolic pressure and the coronary flow were not changed in IMY group relative to controls (Supplemental Table 1). Taken together, the characteristics of myocardia of IMY group confirmed that the isoproterenol treatment induced substantial myocardial injury that presented on day 15 by rhythm disturbances, hemodynamic alterations and specific morphological changes.

Myocytes of the working tissue of the IMY hearts displayed morphology in many aspects similar to that of the control group; nevertheless, they showed increased occurrence of features indicating cellular remodelling (Fig. S1A, B). Large clusters of mitochondria, accumulations of cytosol, increased amounts of rough endoplasmic reticulum and ribosomes were typical. Myofibrils showed increased incidence of branching, misalignment, reduced diameter, and irregular or rugged Z-lines (Fig. S1B). Some regions of cell periphery displayed an increased amount of vesicles and caveolae at the sarcolemma (Fig. S1C). Caveolae-like structures were observed frequently also at T-tubule membranes (Fig. S1D). Increased branching of T-tubules was observed near dyadic microdomains (Fig. S1E), leading to partial fragmentation and to separation of the juxtaposed surface of the SR cisterna from the T-tubule membrane. Longitudinal T-tubules appeared more frequently and formed dyads with terminal cisternae in the vicinity of A-bands (Fig. S1F). Overall, the membrane and contractile system of IMY myocytes looked like under reconstruction, indicating ongoing cellular adaptation.

SUPPLEMENTAL FIGURES

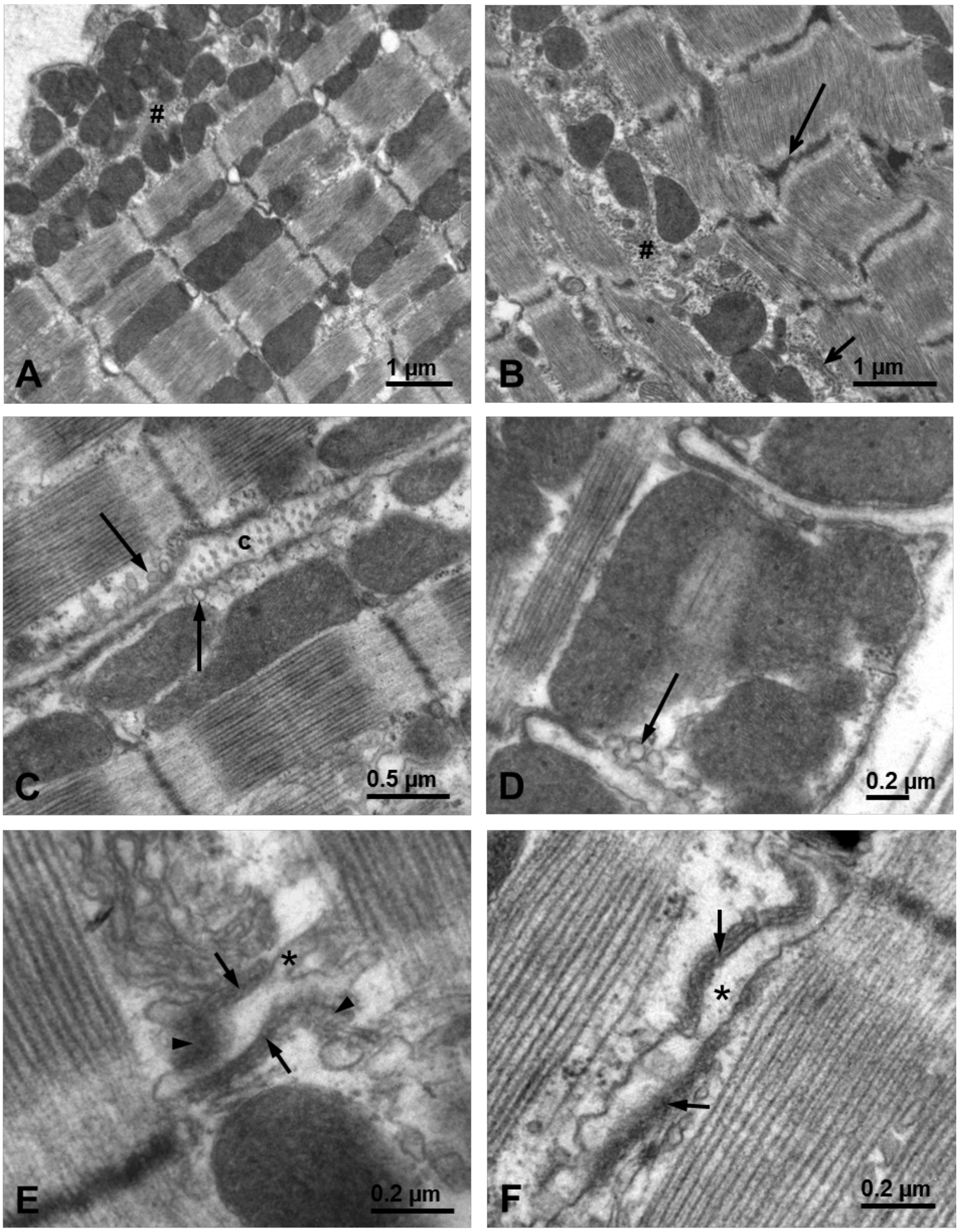

Supplemental Figure S1. Details of myocytes of functional regions of left ventricles of IMY hearts. A – subsarcolemmal zone; # – accumulated cytosol and mitochondria. B – central zone; # – accumulated cytosol; long arrow – Z-lines of distorted myofibrils; short arrow – the endoplasmic reticulum with ribosomes. C – intercellular zone; c – collagen fibres; arrows – plasma membrane rich in caveolae. D – surface zone; arrow – T-tubule with caveolae. E – dyadic microdomain; * – branching T-tubule; arrows – fragmented terminal cisternae; arrowheads – detached terminal cisternae. F – longitudinal T-tubule; * – lumen of T-tubule; arrows – terminal cisternae.

SUPPLEMENTAL TABLES

Supplemental Table S1. Characteristics of hearts in control and IMY rats

	CTR (n = 8)	IMY (n = 9)	P value
**Biometric data**			
Body weight (g)	319 ± 8	284 ± 5	0.0015
Heart weight (g)	1.19 ± 0.03	1.32 ± 0.06	0.072
HW/BW index (mg/g)	3.74 ± 0.11	4.66 ± 0.20	0.0018
LV free wall thickness (mm)	3.26 ± 0.08	3.47 ± 0.11	0.15
***in vivo* ECG data**			
HR (min^{-1})	337 ± 10	398 ± 25	0.042*
R_I amplitude (mV)	0.44 ± 0.04	0.49 ± 0.02	0.20*
R_{II} amplitude (mV)	0.49 ± 0.04	0.33 ± 0.03	0.00064*
S_{III} amplitude (mV)	-0.03 ± 0.02	-0.14 ± 0.03	0.019*
QTc (ms)	53.2 ± 0.6	61.8 ± 2.5	0.015*
***ex vivo* ECG data**			
HR (min^{-1})	210 ± 17	281 ± 23	0.027
R amplitude (mV)	6.25 ± 0.25	9.67 ± 0.44	1.0×10^{-5}
QRS duration (ms)	28.4 ± 0.8	32.4 ± 1.1	0.012
QTc (ms)	55.3 ± 2.2	100 ± 6	3.5×10^{-6}
***ex vivo* functional data**			
LVDP/LVW(mmHg g^{-1})	104 ± 12	50.6 ± 10.7	0.005
LVPd/LVW(mmHg g^{-1})	10.9 ± 1.2	11.5 ± 1.0	> 0.5
CF/HW (ml $min^{-1} g^{-1}$)	7.2 ± 0.4	7.0 ± 0.6	> 0.5

Data are given as mean ± s.e.m.; n – number of animals; P value – Student's two-sample or *paired t-test. HW-heart weight, BW - body weight; HR – heart rate; R amplitudes - amplitudes of R waves in ECGs; QRS - QRS complex; QTc - corrected QT interval $QTc = QT/(\sqrt{(R-R)/200})^2$; LVDP - left ventricular developed pressure; LVPd - left ventricular diastolic pressure; LVW - left ventricular weight; CF - coronary flow.

References

1 Grimm, D. *et al.* Development of heart failure following isoproterenol administration in the rat: role of the renin-angiotensin system. *Cardiovasc Res* **37**, 91-100, doi:10.1016/S0008-6363(97)00212-5 (1998).

2 Kralova, E., Mokran, T., Murin, J. & Stankovicova, T. Electrocardiography in two models of isoproterenol-induced left ventricular remodeling. *Physiol Res* **57 Suppl 2**, S83-89 (2008).

3 Grimm, D., Holmer, S. R., Riegger, G. A. & Kromer, E. P. Effects of beta-receptor blockade and angiotensin II type I receptor antagonism in isoproterenol-induced heart failure in the rat. *Cardiovasc Pathol* **8**, 315-323, doi:10.1016/S1054-8807(99)00021-6 (1999).

4 El-Armouche, A. *et al.* Phosphatase inhibitor-1-deficient mice are protected from catecholamine-induced arrhythmias and myocardial hypertrophy. *Cardiovasc Res* **80**, 396-406, doi:10.1093/cvr/cvn208 (2008).

5 Mikusova, A., Kralova, E., Tylkova, L., Novotova, M. & Stankovicova, T. Myocardial remodelling induced by repeated low doses of isoproterenol. *Canadian journal of physiology and pharmacology* **87**, 641-651, doi:10.1139/Y09-053 (2009).
